# Coexistence and habitat restoration planning for the reintroduction of Spix's macaw

**DOI:** 10.1111/cobi.70105

**Published:** 2025-07-09

**Authors:** Ugo Eichler Vercillo, Silvio Marchini, Matheus Felipe Barbosa Bahia Fritzsons, José Luiz de Andrade Franco

**Affiliations:** ^1^ Center for Sustainable Development University of Brasília Brasília Brazil; ^2^ BlueSky Curaçá Brazil; ^3^ Smithsonian National Zoo and Conservation Biology Institute Front Royal Virginia USA

**Keywords:** biodiversity conservation, Brazil, human–wildlife interactions, protected area, theory of change, área protegida, Brasil, conservación de la biodiversidad, interacción humano‐fauna, teoría del cambio, 生物多样性保护, 人类与野生动物互作, 保护地, 巴西, 变革理论

## Abstract

Spix's macaw (*Cyanopsitta spixii*) is one of the world's most endangered species. Native to the Caatinga of northeastern Brazil—a region marked by significant socioeconomic vulnerability—the species was considered extinct in the wild in 2000. A reintroduction project, however, returned it to its natural habitat in 2022. The long‐term success of this reintroduction hinges on meticulous planning that promotes the coexistence of the birds with the local community and addresses the species’ ecological requirements. This planning should be grounded in evidence derived from both scientific research and local knowledge. Moreover, it must adopt a participatory approach, given its explicit aim to generate benefits not only for the Spix's macaw but also for the human communities sharing its habitat. We devised a participatory planning process aimed at creating and implementing a theory of change for fostering human–Spix's macaw coexistence and restoring the species’ habitat. Drawing on the results of a socioeconomic survey conducted from 2022 to 2023, we convened a workshop in 2024 that brought together representatives from the research, conservation, governmental, and local community sectors. Participants identified the 7 key human–Spix's macaw interactions and considered the positive and negative impacts of these interactions on the macaws and local communities: Caatinga restoration, tourism, extensive livestock farming, hunting and capture, wildlife management, deforestation, and the reintroduction. Fifty‐seven drivers underlying these interactions were identified at the workshop, and participants proposed 51 targeted actions to address these drivers and foster positive changes in the interactions. The outcomes of the workshop are intended to guide local territorial development centered on conservation to contribute to a more sustainable future for one of the most emblematic species in global biodiversity conservation.

## Abstract (es)

Coexistencia y planeación de la restauración de hábitat para la reintroducción para el guacamayo de Spix

**Resumen:**

El guacamayo de Spix (*Cyanopsitta spixii*) es una de las especies más amenazadas del mundo. Originaria de la Caatinga del noreste de Brasil – una región marcada por una importante vulnerabilidad socioeconómica – la especie fue considerada extinta en estado silvestre en el 2000. Sin embargo, un proyecto de reintroducción la devolvió a su hábitat natural en 2022. El éxito a largo plazo de esta reintroducción depende de una planificación meticulosa que promueva la coexistencia de las aves con la comunidad local y atienda los requisitos ecológicos de la especie. Esta planificación debe basarse en pruebas derivadas tanto de la investigación científica como de los conocimientos locales. Además, debe adoptar un enfoque participativo, dado su objetivo explícito de generar beneficios no sólo para el guacamayo de Spix, sino también para las comunidades humanas que comparten su hábitat. Diseñamos un proceso de planificación participativa destinado a crear y aplicar una teoría del cambio para fomentar la coexistencia entre humanos y guacamayos de Spix y restaurar el hábitat de la especie. A partir de los resultados de una encuesta socioeconómica realizada entre 2022 y 2023, convocamos un taller en 2024 que reunió a representantes de los sectores de la investigación, la conservación, el gobierno y la comunidad local. Los participantes identificaron las siete interacciones clave entre humanos y guacamayos de Spix: restauración de la Caatinga, turismo, ganadería extensiva, caza y captura, gestión de la fauna, deforestación y reintroducción. Después consideraron los impactos positivos y negativos de estas interacciones sobre los guacamayos y las comunidades locales. En el taller se identificaron 57 factores subyacentes a estas interacciones y los participantes propusieron 51 acciones específicas para abordar estos factores y fomentar cambios positivos en las interacciones. Los resultados del taller pretenden orientar el desarrollo territorial local centrado en la conservación para contribuir a un futuro más sostenible de una de las especies más emblemáticas en la conservación de la biodiversidad mundial.

## 斯比克斯鹦鹉重引入项目的共存与栖息地恢复规划

**【摘要】** 斯比克斯鹦鹉(*Cyanopsitta spixii*)是世界上最濒危的物种之一。该物种原产于巴西东北部的卡廷加地区(一个社会经济十分脆弱的区域), 已在2000年被认为野外灭绝。然而, 2022年, 一个重引入项目将该物种重新引入了其自然栖息地。该项目的长期成功依赖于周密的规划, 这样的规划需要促进该物种与当地社区的共存, 并满足该物种的生态需求。规划应基于科学研究与本地知识相结合的证据。此外, 考虑到该项目的明确目标是为斯比克斯鹦鹉和共同使用其栖息地的人类社区同时带来收益, 项目规划必须采用参与式方法。我们设计了一个参与式规划过程, 旨在制定并实施一个促进人类与斯比克斯鹦鹉共存并恢复该物种栖息地的变革理论。基于2022‐2023年进行的社会经济调查结果, 我们在2024年举办了一个研讨会, 聚集了来自研究、保护、政府等领域及当地社区的代表。参与者确定了七个人类与斯比克斯鹦鹉互作的关键领域, 包括卡廷加生态系统恢复、旅游业、大规模畜牧业、狩猎与采集、野生动物管理、森林砍伐以及重引入, 并分析了这些互作对鹦鹉和当地社区的正面和负面影响。在研讨会上, 参与者确定了互作的57个驱动因素, 并提出了51项针对性行动, 以应对这些驱动因素并促进互作的积极变化。研讨会的成果可用于指导以保护为中心的地方区域发展, 从而推动全球生物多样性保护中最具代表性的物种之一的更可持续性未来。**【翻译:胡怡思;审校:聂永刚】**

## INTRODUCTION

Spix's macaw (*Cyanopsitta spixii*) is an endemic parrot of the Brazilian Caatinga domain, is confined to Bahia state, and is currently classified as extinct in the wild by the International Union for Conservation of Nature (IUCN) (BirdLife International, 2024). The last individual, a male, was found in 1990 and monitored. After October 2000, it was not detected again (Juniper, 2002). The species became extinct in the wild primarily due to poaching and habitat loss (Lugarini et al., 2021). A seminal conservation effort in 2022 led to the release of 20 Spix's macaws in 2 designated protected areas: Spix's Macaw Refuge and Spix's Macaw Environmental Protection Area. Both areas are in the municipalities of Juazeiro and Curaçá, Bahia (Purchase et al., 2024). Following a population viability analysis (PVA), a sustained release strategy was recommended: a minimum of 10 and up to 20 birds released per year over 20 years. The releases were to be complemented by an ambitious restoration initiative targeting a minimum of 15,000 ha of riparian forest in the protected areas (Vercillo et al., 2024, 2023).

Decades of unsustainable land use, particularly livestock management, have degraded Caatinga vegetation, and this degradation has been exacerbated by climate change (Silva et al., 2019, 2017). Spix's macaw conservation must aim to mitigate the threats and reinstate ecological conditions conducive to wild population establishment, including assisted adaptation to address initial challenges, such as predation risk and food and shelter deficiencies (Carver, 2016; IUCN/SSC, 2013; White Jr. et al., 2012). These actions could help conserve and restore Caatinga habitat in general and promote inclusive and sustainable socioenvironmental development to support the vulnerable human populations that share this unique habitat (Purchase et al., 2024).

The presence and work of external actors in the region introduce additional complexities, because of the lack of trust, that could lead to conflicts (Young et al., 2016). The socioeconomic status of local people presents a significant challenge for conservation, particularly concerning the reintroduction of Spix's macaw.

Therefore, the adoption of an integrative, multistakeholder approach to species conservation planning could improve outcomes for some of the most challenging cases. Science‐based, participatory approaches to planning can create a turning point for threatened species when stakeholders move quickly to determine effective ways of working together. Lees et al. (2021) found that over 13 years, none of 35 species in 23 countries with conservation plans became extinct. Simulated counterfactual projections indicated that without these conservation interventions, approximately 8 species would have gone extinct during that period.

Conservation plans and management and policy decisions concerning human–wildlife interaction have traditionally addressed the negative impacts of human activities on endangered wildlife from an ecological perspective (Frank, 2015; IUCN/SSC, 2013). However, planning for large‐scale coexistence entails considering the entire spectrum of interactions between humans and wildlife and considering what stakeholders perceive as impacts of interactions that warrant attention (Marchini et al., 2021). These impacts can be negative or positive and range from tangible effects, such as loss of livestock and income, to intangible effects, such as fear or happiness, and they affect both people and wildlife. A coexistence approach should aim for a balance among economic growth, community well‐being, and environmental protection (Caro & O'Doherty, 1999; Hilty et al., 2006).

The concept of territorial development based on conservation (TDBC) lies in an ecosystemic vision of nature conservation—that is, conservation is not only a technical or biological process but also a political, social, and cultural one. Moreover, it presupposes that conservation instruments should operate across different scales by including local communities and their activities. In other words, conservation should be integrated into broader development projects to encourage sustainable development practices that respect ecological limits and minimize negative environmental impacts (Martins, 2018; Nora, 2022).

To address the challenge of promoting the coexistence between Spix's macaw and the local community, we conducted a socioeconomic assessment of the local community and devised a process of planning for human–macaw coexistence that explicitly acknowledges the multifaceted nature of the issue. Our approach differs from traditional methods of conservation planning and managing negative interactions, such as human–wildlife conflicts, due to its broad purpose and decision‐making foundation. The process is different because it prioritizes coexistence as its goal, focuses on systemic thinking, and emphasizes verifiable change (Marchini et al., 2021). This holistic and useful framework seeks not only to address immediate conflicts but also to foster long‐term sustainable relationships between humans and wildlife through measurable and systemic strategies (Reed et al., 2019) with the goal of supporting the Spix's macaw reintroduction project and promoting reforestation of native vegetation.

## METHODS

### Study site

The study was conducted in the 2 Spix's macaw protected areas north of Bahia State, Brazil, in the municipality of Curaçá (9°9ʹ55.44ʺ S, 39°46ʹ43.08ʺ W). The study was established by the Chico Mendes Institute for Biodiversity Conservation, the governmental body responsible for managing the protected areas (authorization SISBIO 85922‐1/2022). We complied with all requirements of the Chico Mendes Institute in our work, including obtaining prior authorization for access to communities, and signed free, prior, and informed consent forms from participants.

Recorded occupation of Curaçá by the Indigenous Tapuia dates to 1562. The municipality was formally established in 1832 as Pambu and was renamed Curaçá in 1890. Historically, the local economy revolved around livestock management and wood extraction. Recently, irrigated fruit production was established. The city encompasses several districts, and recent demographic data from the 2022 census indicate a population of 34,180 individuals with a density of 5.74 inhabitants per square kilometer (IBGE, 2024; Lopes, 2000; Lopes Gonçalves, 2004).

The 2024 Social Economic Assessment of the Brazilian Institute of Geography and Statistics indicates Curaçá faces significant economic and educational challenges, as evidenced by its low per capita gross domestic product per capita of approximately US$2,000 in 2021, which is substantially below the national average of around $8400 (IBGE, 2024). The region has limited wealth generation as a result of a low employment rate of 6.96% in 2022 (national average 47.8%) (IBGE, 2024). Further, 52% of Curaçá’s population earns less than half the minimum wage per capita. Nationally, 28.7% earns less than half that (IBGE, 2024). The school enrollment rate of 97.4% for children aged 6–14 is slightly below the national average of 99.7% (IBGE, 2024). The basic education development index scores for both initial (4.7) and final years (4.1) in Curaçá fall short of the national averages of 5.9 and 4.9, respectively (IBGE, 2024).

### Socioeconomic survey and data analyses

We conducted structured interviews from 2022 to 2023 with property owners or occupants in the protected areas. The interviewers visited every residence occupied in the study area. We used Kobotoolbox (2024) to facilitate data collection and organization, and all data were anonymized and aggregated before analyses. All interviews were carried out in person and conducted by the same team of interviewers who had extensive knowledge of the local community. Interviews were conducted in Portuguese with a standard questionnaire. One person was interviewed per property without others present to ensure the independence of reported data and avoid interference from others. Interview questions are in Appendix S1.

The variables that characterize the population were summarized by group. Information was collected about the owner, the property, economic uses of the land, material goods, household characteristics, and perception of the macaw conservation project and major environmental issues. The cluster analysis was conducted using binary variables representing key economic land uses and personal assets.

For statistical analyses, we used the Statistical Package for Social Sciences (SPSS, 2008). We used a cluster analysis to identify distinct groups in the community. We used a hierarchical model of Jaccard distance for binary variables, which allowed for the partitioning of a heterogeneous population into more homogeneous subgroups based on similarities among elements. This approach facilitated the segmentation of data and the identification of groups with shared characteristics, such as patterns of land use and resource possession, and thus enhanced comprehension of the community dynamics (Doni, 2004; Jain et al., 1999).

### Theory of change

In February 2024, a coexistence workshop with local stakeholders was held that followed the method developed by Marchini et al. (2021). Thirty local stakeholders from diverse backgrounds—including community representatives, farmers’ unions, nongovernmental organizations, government institutions, researchers, and the BlueSky reforestation company—devised a conceptual framework to examine the dynamics between human communities and Spix's macaw reintroduction project.

The primary focus was to catalog direct and indirect interactions associated with the Spix's macaw and the habitat recovery efforts. During the workshop, the group of participants identified and classified 10 major interactions between humans and Spix's macaw and assessed their impacts as either positive or negative on humans and the birds. As an output, a theory of change, an action plan, and a monitoring matrix to track progress were developed.

## RESULTS

### Socioeconomic survey

**FIGURE 1:**
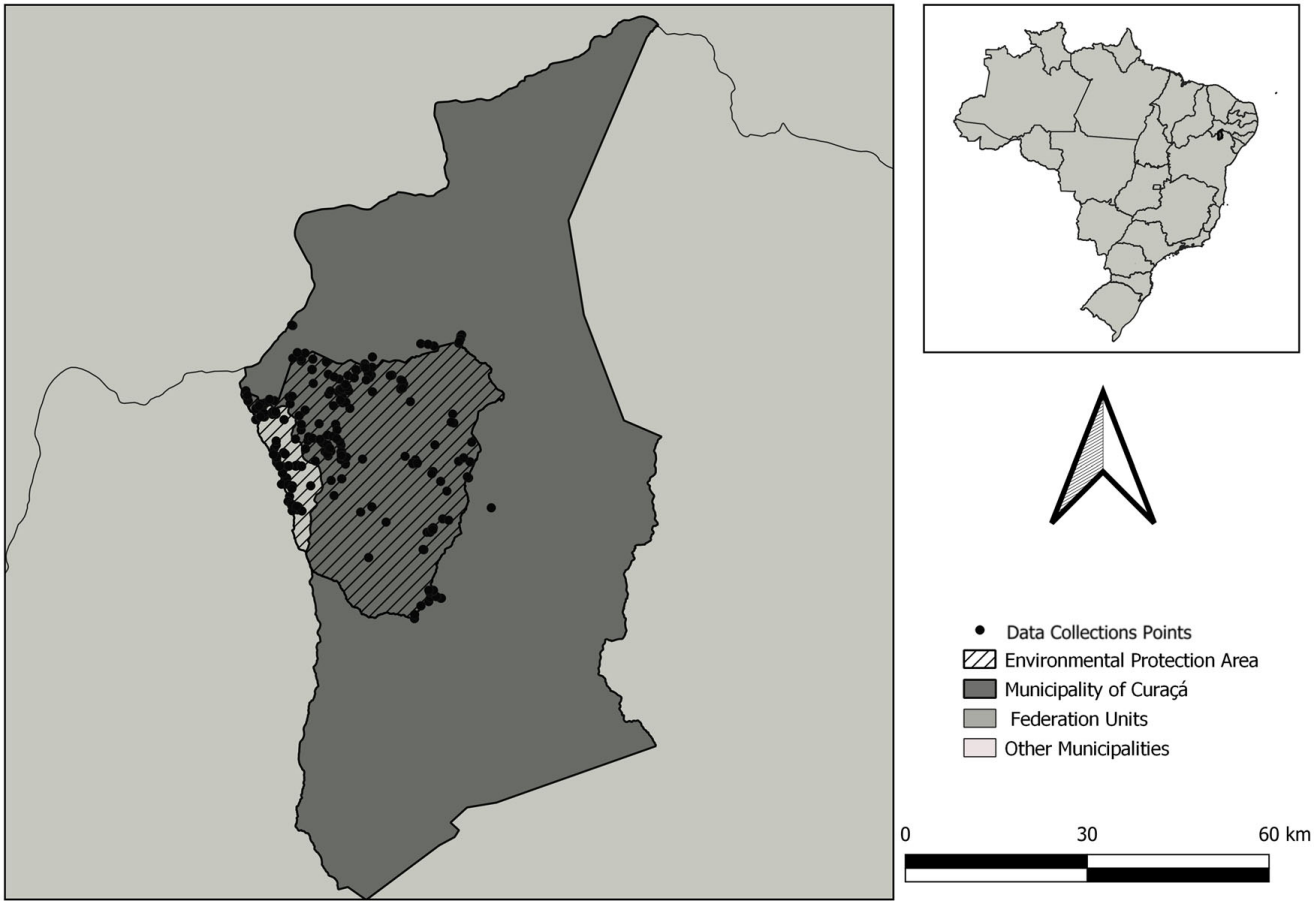
Study area and data collection locations for the socioeconomic survey conducted in the context of the Spix's macaw (*Cyanopsitta spixii*) reintroduction project in the state of Bahia, Brazil (dark gray, Municipality of Curaçá; hatching, Spix's Macaw Refuge and Environmental Protection Area; black dots, locations where household interviews were conducted to collect socioeconomic, land use, infrastructure, and environmental perception data from local residents).

From the 1790 properties identified in the 2022 census, 437 farms were visited, and 288 completed the entire questionnaire (Figure 1). The majority of respondents were men (72%). The sample size provided 95% accuracy (error <5.0%) (Bussab & Morettin, 2006).

**FIGURE 2:**
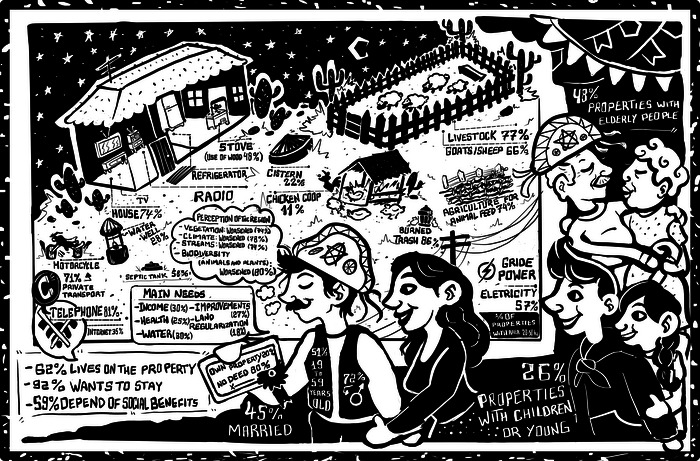
Socioeconomic characteristics of rural properties in the municipality of Curaçá, Bahia, derived from structured interviews conducted across 288 registered landholdings. The figure was developed as part of a strategy for communicating research findings to the local population and is presented in the traditional cordel style, a culturally relevant visual language in the Caatinga region.

We found a high level of poverty. Many families depended on social programs, such as the Bolsa Família (a financial support program financed by the Brazilian government to alleviate poverty and reduce inequality), pensions, and crop insurance for their livelihood. Among the 288 respondents, 41.3% received pensions, 14.6% were enrolled in Bolsa Família, 3.1% received crop insurance, and 35.1% did not receive any social benefits. Property sizes varied: 16.3% were ≤10 ha, 25.7% were 21–50 ha, and 3.1% were >500 ha. Most respondents (80.2%) lacked formal property titles, although 56.6% had some form of documentation (Table 1; Figure 2).

**TABLE 1:** Socioeconomic aspects and environmental understanding of the rural community of Curaçá, Brazil (human population 288).

Category	Attribute	Percentage (%)
User profile	Male	72.0
	Married	45.1
	Properties with people from 19 to 59 years old	51.2
	Properties with elderly people	48.3
	Properties with children or young people	26.5
	Dependent on social benefits	59.0
	Land squatter	80.9
	Born on the property	62.5
	Tendency to remain in the region	92.7
	Main transport private	71.2
Property characteristic	Property used as residence	62.6
	Property used for livestock	77.8
	Property used for agriculture	20.1
	Property without title deed	80.2
	Property with ownership documentation	56.6
Improvements	House	74.0
	Pigsty	68.4
	Chicken coop	11.5
Asset	Refrigerator, stove, TV, radio, motorcycle	N/A
Infrastructure	Septic tank	58.3
	Water source artesian well	28.5
	Water source consumption cistern	22.2
	Electricity via general grid	57.6
	Waste disposal burning	86.8
	Telephone access	81.9
	Internet access	35.1
Other activities	Charcoal or firewood extraction	48.3
	Agriculture for animal feed	74.0
	Animal husbandry goats	66.7
	Animal husbandry sheep	60.4
Community main need	Income	30.0
	Water	30.0
	Property improvements	27.0
	Health	25.0
	Land tenure regularization	18.0
Perception of the environment status	Vegetation deterioration	74.7
	Climate deterioration	78.1
	Stream deterioration	74.7
	Biodiversity deterioration	80.9
Understanding of environmental concepts	Biodiversity heard about before	61.5
	Climate change heard about before	69.4
	Extinction heard about before	69.4
	Carbon credits introduced by project	68.1
	Global warming heard about before	69.1
	Land leasing heard about before	53.8

Residences were 62.6% of properties, and 77.8% of respondents engaged in livestock farming and 20.1% in agriculture.

Goats were raised on 66.7% of properties and sheep on 60.4%. Water access was primarily through artesian wells (28.5%) and cisterns (22.2%). Septic tanks were used on 58.3% of properties. Electricity was available in 63.9% of properties, mainly supplied through the electrical grid (57.6%). Most households (86.8%) burned their waste, and 48.3% used charcoal or firewood for cooking. Telecommunication access was high (81.9% had telephones), but only 35.1% had internet access (Table 1; Figure 2).

Key needs identified included increased income and access to water (30%), property improvements (27%), increased health services (25%), and land tenure regularization (18%). Respondents perceived a decline in local vegetation, climate, water bodies, and biodiversity over recent years. Awareness of environmental concepts was relatively high; over 60% were familiar with environmental terms, such as *biodiversity*, *climate change*, and *global warming*. However, 68.1% had not heard of carbon credits (Table 1; Figure 2).

The cluster analysis results were not statistically significant due to insufficient correlation among the selected variables, which is essential for assessing model adherence. As a result, the generated clusters were based on the distance to the mean of the variables rather than meaningful correlation between them. This indicates that although the groups shared similarities in average variable values, these similarities lacked statistical correlation, suggesting that the clustering pattern was a random characteristic of the dataset.

### Theory of change

**FIGURE 3:**
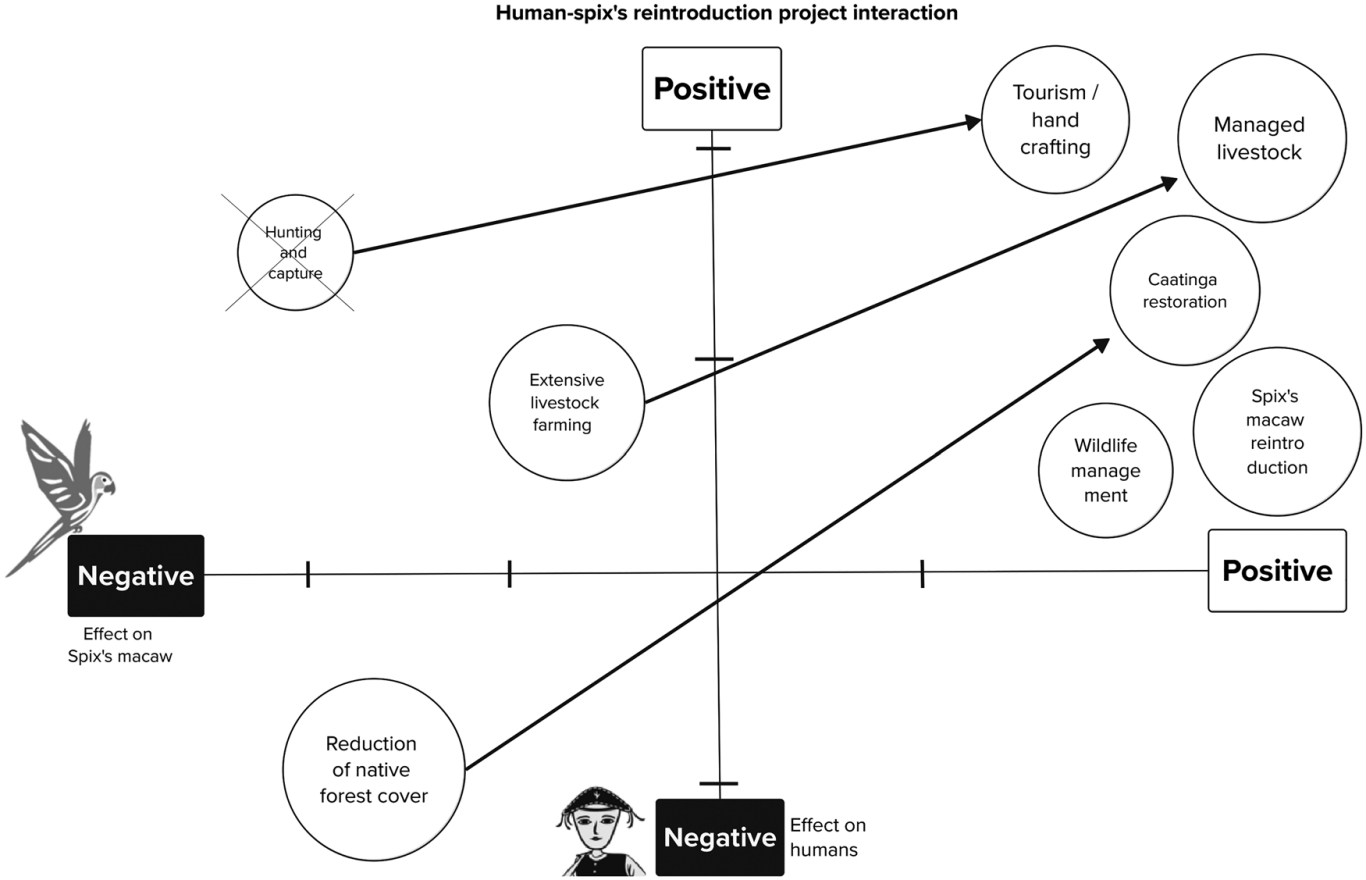
Direct and indirect interactions associated with Spix's macaw and efforts to restore its habitat (arrows, expected shift; x, interaction [hunting] should be avoided).

Figure 3 provides a summary of the interactions between humans and the Spix's macaw reintroduction project. Human activities had mixed effects on the species and local communities. Negative drivers, such as hunting and extensive livestock farming, directly threatened Spix's macaw, and native forest loss negatively affected both species viability and human livelihoods. In contrast, positive actions, including managed livestock systems, caatinga restoration, wildlife management, and alternative income sources (e.g., tourism and handicrafts), mitigated these pressures and contributed to sustainable coexistence. Figure 3 shows how targeted management interventions can reduce threats and promote positive human–wildlife interactions.

**FIGURE 4:**
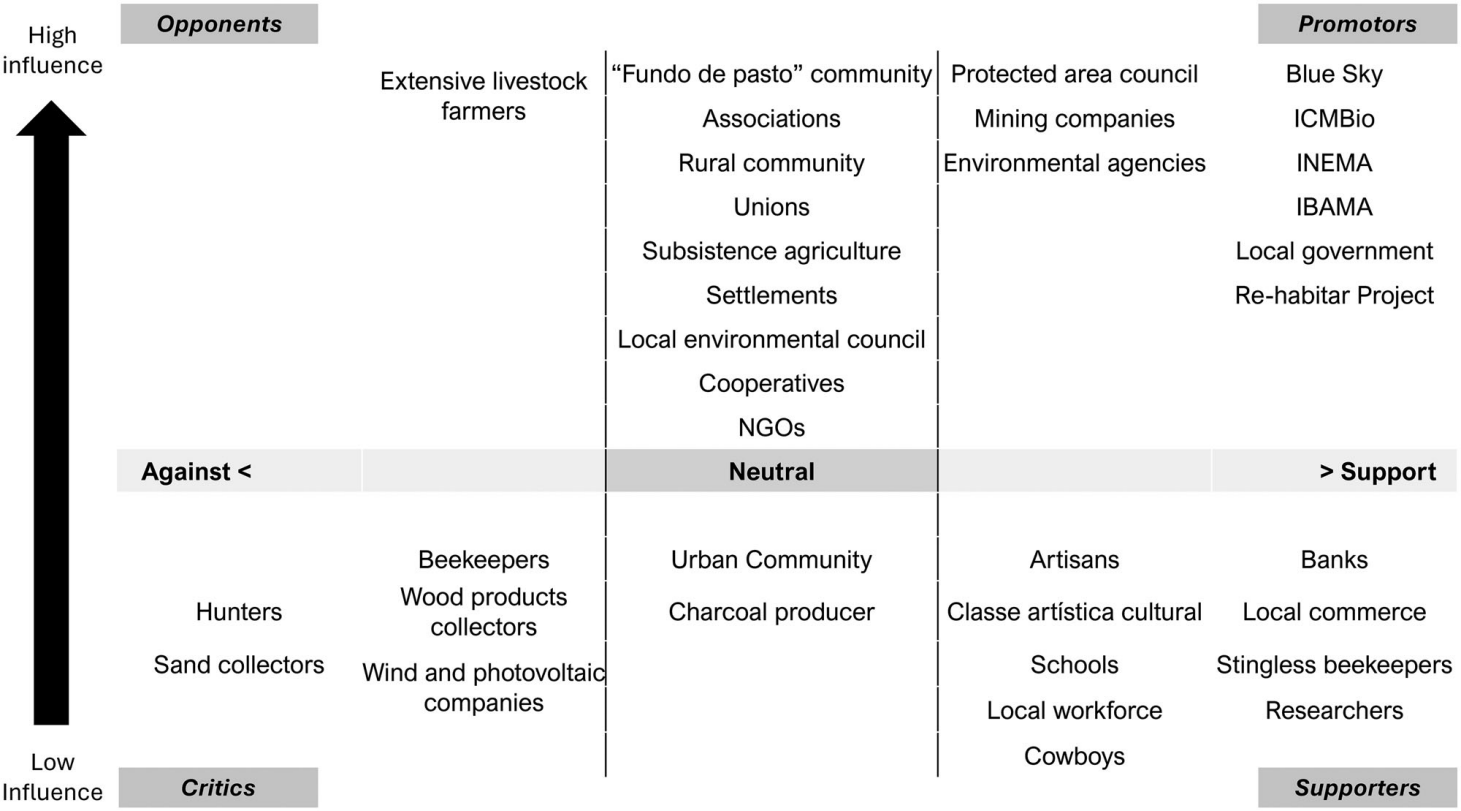
Stakeholder mapping from the theory of change workshop conducted to support the Spix's macaw (*Cyanopsitta spixii*) reintroduction planning. Participants were categorized based on their perceived opinion of the project to allow identification of actors for engagement strategies.

To move interactions toward the goal of coexistence (Figure 3), particularly to mitigate illegal activities, such as hunting, the strategic plan developed in the workshop identified key stakeholders and classified them as promotors (9 people), supporters (9), critics (5), and opponents (1) (Figure 4). Eleven stakeholders were classified as neutral. Strategic actions tailored to engage these groups effectively were delineated and included capacity building for sustainable land use, provision of legal and technical support for land regularization, and increased community participation and economic development. There actions were aimed at harnessing the positive influences of promotors and supporters, mitigating criticism, neutralizing opposition, and convincing the neutral stakeholders to be supporter or promotor.

**FIGURE 5:**
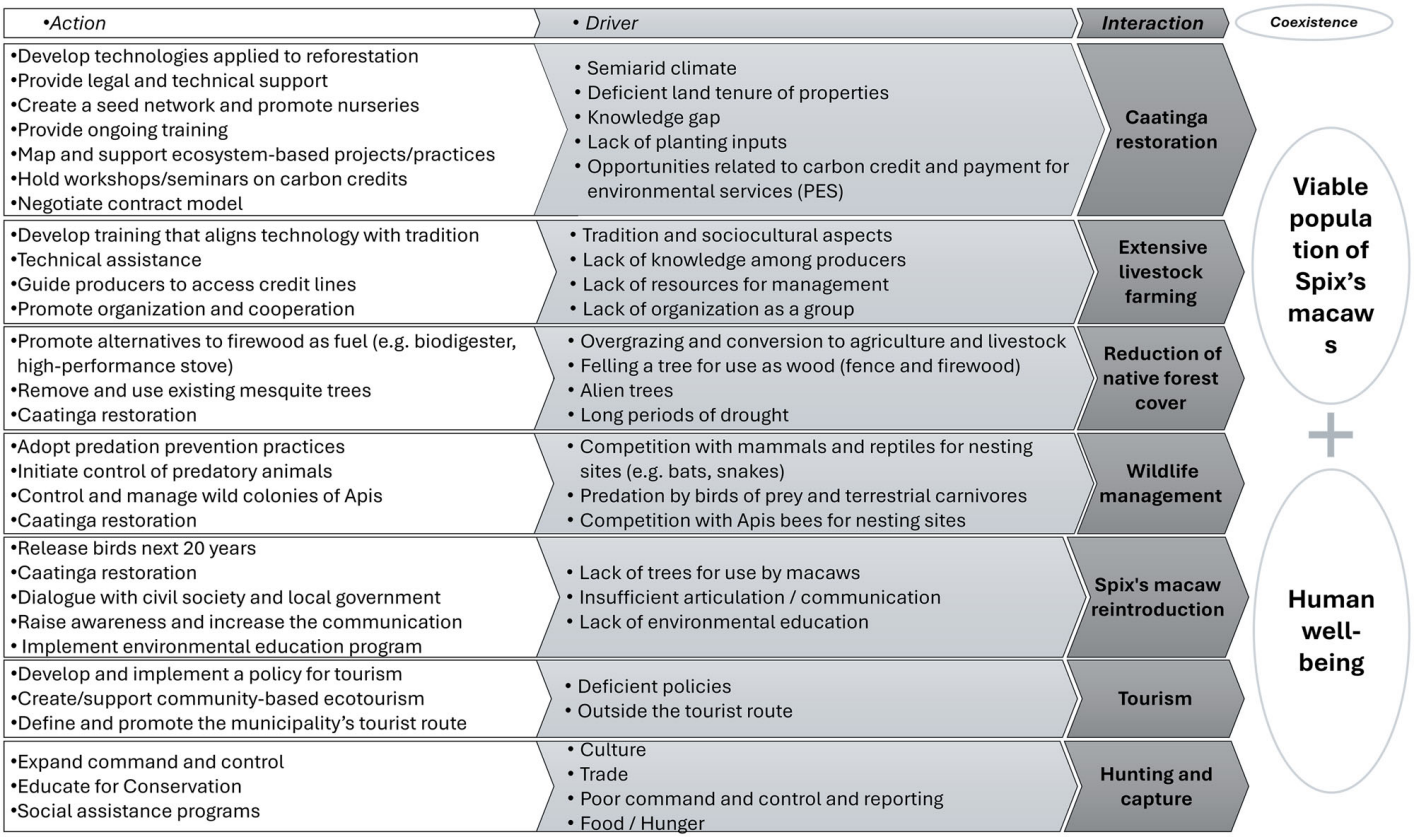
Theory of change showing the interactions and factors shaping the coexistence between humans and reintroduced Spix's macaws in the wild (dark gray, human–Spix's macaw interactions; light gray, factors that either intensify negative interactions or hinder positive ones; white boxes, actions designed to mitigate the effects of these drivers and promote coexistence). The full diagram with the relations is in Appendix S2.

Participants established a 10‐year timeline to achieve the desired objectives for change (Appendix S2). They formulated 49 actions to address the 7 listed interactions (Figure 5). Interventions were identified that were expected to bring about the desired changes, either directly or indirectly. To monitor these interventions, a tracking matrix was developed, incorporating specific indicators and verification methods (Appendix S3). This matrix was designed for use in assessing the progress and effectiveness of the implemented actions over the course of the decade‐long initiative.

## DISCUSSION

### Socioeconomic survey

Our socioeconomic assessment showed a strong connection between the local community and the environment, with families expecting to spend their entire lives in Curaçá. However, the community's vulnerability is a threat to Spix's macaw reintroduction and habitat restoration project (Duraiappah, 1998) because socioeconomic vulnerability often leaves people few options other than exploiting natural resources, potentially leading to hunting, poaching, and unsustainable harvesting or land use.

Our results highlight a significant socioeconomic vulnerability, with Curaçá’s per capita income and other socioeconomic indicators falling below national and state averages. Thus, a conservation strategy must include actions to reduce the local community's vulnerabilities to avoid poaching and deforestation activities. This means working with relevant parties to increase employment, promote development of sustainable economic activities related to land use, and provide essential services (i.e., access to piped water, municipal sewage systems, and land ownership documentation).

### Theory of change

The survey results aligned with the theory of change workshop outcomes, which highlighted the need for opportunities for families and degradation of natural areas. The theory of change workshop emphasized the negative impact of certain activities on the reintroduction of Spix's macaw and identified the need for best practices in livestock management to reduce overgrazing on the Caatinga and implement efficient management processes.

The degraded Caatinga requires strong actions for recovery, including reforestation projects (*recaatingamento* in Portuguese). These integrated efforts can increase local community engagement, strengthen relationships between promoters and supporters, and shift neutral, critical, and opposing positions in favor of the project. Alternative strategies, such as tourism, handicraft sales, and reforestation, present potential opportunities and should be incentivized by local organizations and the government, which aligns with the Spix's macaw reintroduction project.

The workshop outputs underline the need for adaptive planning and research focusing on changes that benefit both wildlife and human communities. This involves understanding the system's complexity and how it responds to management actions and emphasizing equitable distribution of costs and benefits among different stakeholder groups. Changing the paradigm of human–wildlife relationships and reducing rural community vulnerability, as proposed by Duraiappah (1998), Roe (2006), and Carver et al. (2021), is essential.

Our approach facilitated the identification of interactions and the formulation of strategies to mitigate conflicts while amplifying positive outcomes. The workshop culminated in defining specific outputs and indicators, pivotal for monitoring the action implementation phase. This comprehensive approach underscores the project's commitment to fostering harmony between conservation efforts and community interests.

### Territorial development based on conservation

The transdisciplinary approach we employed integrated ecological and social sciences to develop and refine specific pathways for conservation action and emphasized stakeholder engagement, adaptive management, and evidence‐based decision‐making. Our comprehensive strategy seeks not only to protect and enhance Spix's macaw populations but also to establish a coexistence framework that benefits both wildlife and human communities within the Spix's Macaw Protected Areas.

This approach, known as TDBC, provides a foundational governance framework that reconciles conservation and development agendas across scales, sectors, and disciplines. By bridging the gap between global policy frameworks and local realities, as highlighted by Reed et al. (2019), the TDBC ensures that international conservation strategies are grounded in practical, context‐specific solutions. Effective conservation of Spix's macaw requires a delicate balance among livestock farming, native vegetation restoration, and active engagement with local communities.

The coexistence framework between humans and Spix's macaw can be significantly strengthened through the application of the TDBC. By aligning livestock management and land‐use practices with the goals of caatinga reforestation, this approach promotes sustainable land management practices that support conservation efforts while fostering local economic development. Additionally, leveraging global interest in Spix's macaw can catalyze local development initiatives, such as habitat restoration, ecotourism, and the sale of handcrafted goods, that can create synergistic benefits for both conservation and livelihoods.

## AUTHOR CONTRIBUTIONS

Ugo Eichler Vercillo conceived and conducted the study. Silvio Marchini was responsible for the theory of change. Matheus Felipe Barbosa Bahia Fritzsons assisted with the use of Kobotoolbox and analyzed the survey data. Ugo Eichler Vercillo and José Luiz de Andrade Franco led the writing of the manuscript. All authors contributed critically to the drafts and gave final approval for publication.

## Supporting information



Supporting Information

Supporting Information

Supporting Information
