# Supplementary material for: Coexistence and habitat restoration planning for the reintroduction of Spix's macaw
Source: Conserv Biol. 2025 Jul 9;39(6):e70105. doi: 10.1111/cobi.70105 (PMC12658936; doi:10.1111/cobi.70105)
Supplement: Supplementary file 1 — Supporting Information [file COBI-39-e70105-s002.pdf]

COEXISTENCE AND HABITAT RESTORATION PLANNING FOR THE REINTRODUCTION OF THE SPIX'S MACAW - Appendix S1

Socioeconomic Survey Questionnaire

0.0. Interviewer's Name

0.1. Interview Location Coordinates

0.2. Do you agree to participate in the survey?

0.2.1. Has the Consent Form been signed?

0.3. What is the Sector?

Respondent Information

1.1. Name of the Occupant Interviewed

1.2. Is the respondent the owner or squatter?

1.3. Respondent's Phone Number

1.4. Residential Address for Correspondence

Owner Information

2.1. Name of the Owner or Squatter

2.2. Owner's CPF (Taxpayer Registry Number)

2.4. Is the signature notarized?

2.5. Gender

2.6. Respondent's relationship to the property

2.7. Main Occupation

2.8. Owner's Contact Information

2.9. Marital Status

2.10. Spouse's Name

2.11. Spouse's CPF

2.12. Number of people residing on the property

Property Information

3.1. Name of the Property

3.2. Current Status of the Property

3.2.1. Does it have a deed?

3.3. Does the property have CAR/CEFIR (Environmental Rural Registry)?

3.4.1. Do you have any land ownership documentation for the property?

3.4.2. How long have you held possession of the property?

3.4.3. Is there any lease agreement with other individuals or companies?

3.5. Describe any subdivision of the property

3.6. Total Area of the Property

3.7. Simplified Deed Description

3.8. Property Polygon (Geographical Shape)

3.9. Boundary Observations (Include information on land disputes, if any)

3.10. Do you have employees?

Property Use and Infrastructure

4.1. Main Land Use(s) – General Uses

4.3. Describe Existing Improvements (e.g., constructions, facilities)

Production Information

5.1. Is there production of any of the following? (Corn, Beans, Cassava, Watermelon, Pumpkin, Coriander, Animal Feed)

5.2. Do you raise any of the following animals? (Goat, Sheep, Cattle, Donkey, Horse, Chicken, Beekeeping)

5.3. Extraction of Non-Timber and Timber Products

5.5. Do you have authorization for vegetation suppression?

5.6. Is the property fenced?

5.7. Describe the fenced areas

5.8. Characterize the areas designated for pasture

5.8.1. Size of the fenced areas

Income Information

6.1. What is the monthly income from selling products produced on the property?

6.2. Is there income from social programs? Which programs? What is the monthly amount?

6.3. Are there other sources of income? What are they and what is the amount?

6.4. What is the total family income? (monthly)

6.5. Do you have a bank account?

Household Infrastructure

7.1. What is the main type of Sanitary Sewage System?

7.2. What is the main source of Water Supply?

7.3. Do you have Electricity?

7.4. What fuels are used for cooking in your household?

7.9. Which household appliances do you have?

7.10. Waste Disposal Method

Community and Residency Information

9.1. Are you a member of any residents' or producers' association?

9.2. How many years have you lived in this municipality?

9.3. Do you plan to move or remain in the region?

Transportation and Public Services

10.1. What is your primary means of transportation?

10.2. Does the public transport system meet your needs?

Health Services

11.1. When was the last time you or a family member visited a doctor?

11.2. When was the last time you or a family member visited a dentist?

11.3. Are you monitored by a community health agent?

Education and Connectivity

12.1. What is the distance to the nearest school?

12.2. How do children travel to school?

12.4. Do you have internet access on the property?

12.4. Do you have telephone access on the property?

12.5. What is the greatest need for you and your family at this moment?

12.6. What is the greatest need related to your income?

Perceptions and Environmental Awareness

13.1. What is your perception of changes in vegetation over the past 10 years?

13.2. What is your perception of changes in climate over the past 10 years?

13.3. What is your perception of changes in streams over the past 10 years?

13.4. What is your perception of changes in plants and animals over the past 10 years?

13.5. What are the main threats to the caatinga biome?

Knowledge and Project Perception

14.1. Are you familiar with the Spix's macaw?

14.2. Have you heard about Biodiversity?

14.3. Have you heard about Climate Change?

14.4. Have you heard about Global Warming?

14.5. Have you heard about Extinction?

14.6. Have you heard about Carbon Credits?

14.7. Have you heard about Rural Land Leasing?

14.8. How do you think the project could benefit you and your family?

14.9. What concerns you most about the Spix's macaw reintroduction project?
