# Supplementary material for: Coexistence and habitat restoration planning for the reintroduction of Spix's macaw
Source: Conserv Biol. 2025 Jul 9;39(6):e70105. doi: 10.1111/cobi.70105 (PMC12658936; doi:10.1111/cobi.70105)
Supplement: Supplementary file 2 — Supporting Information [file COBI-39-e70105-s001.pdf]

COEXISTENCE AND HABITAT RESTORATION PLANNING FOR THE REINTRODUCTION OF THE SPIX'S MACAW - Appendix S2

Diagram of the Theory of Change illustrating the interactions and factors shaping the coexistence between humans and reintroduced Spix's macaws in the wild. Human-Spix's macaw interactions are represented in purple, while the drivers, shown in yellow boxes, are factors that either intensify negative interactions or hinder positive ones. The actions, depicted in green boxes, are designed to mitigate the effects of these drivers and promote coexistence

COMPLETE DIAGRAM OF THEORY OF CHANGE

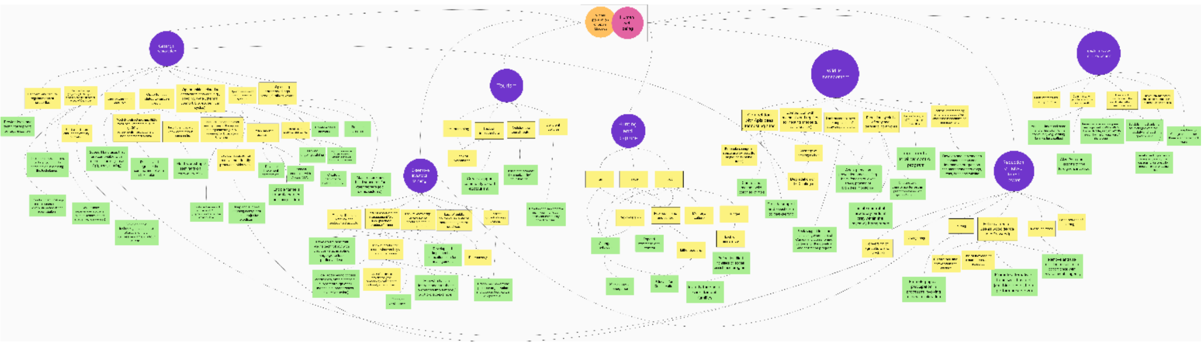

INTERACTIONS

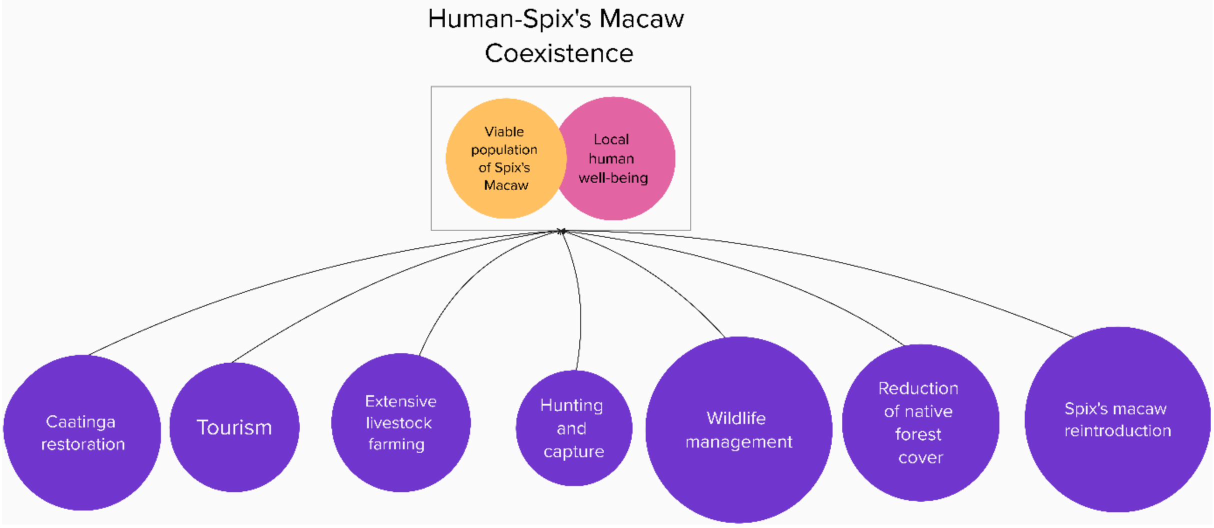

DRIVERS AND ACTIONS OF EACH INTERACTION

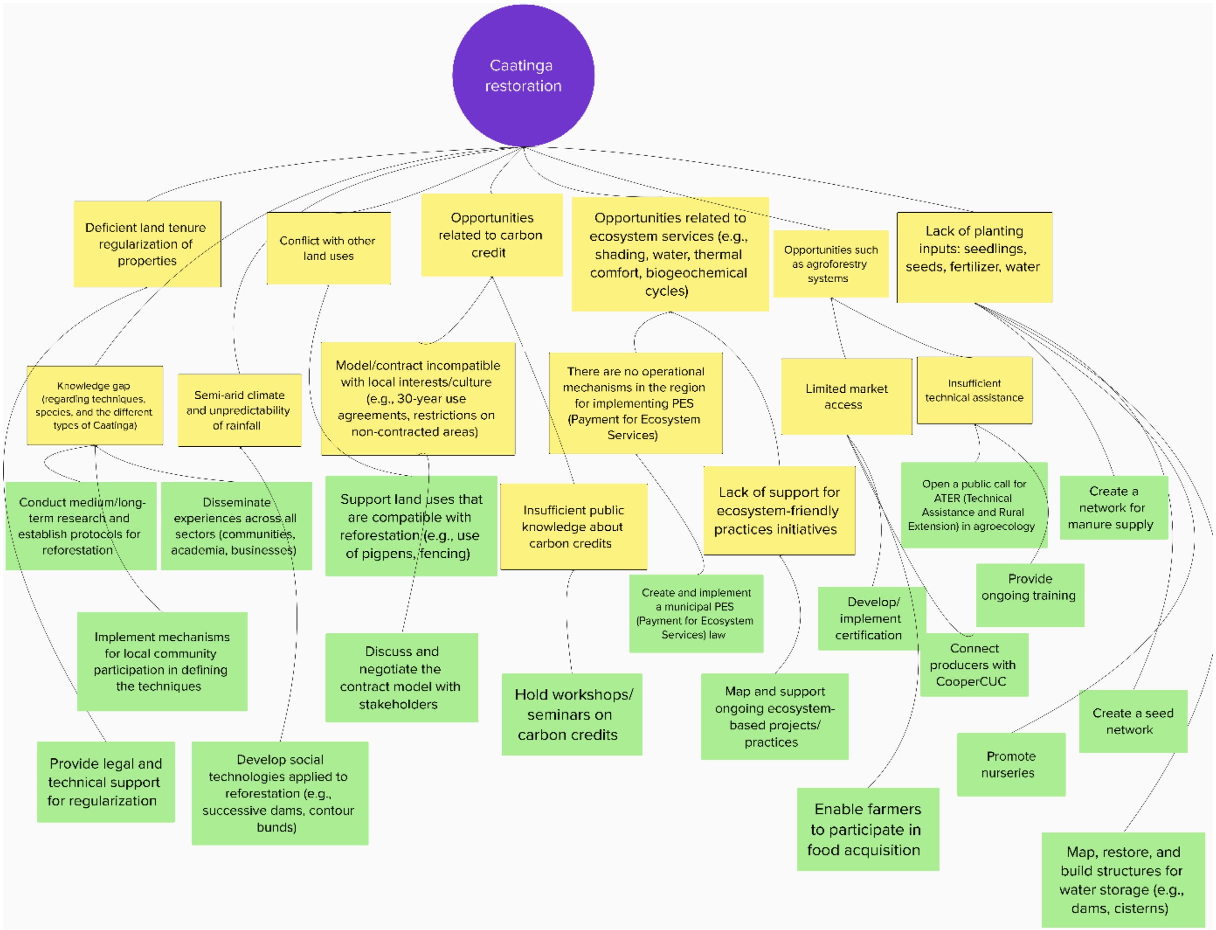

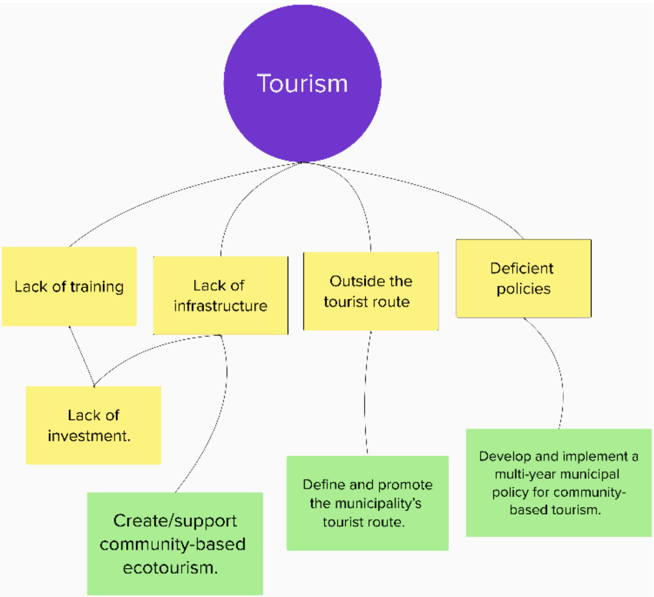

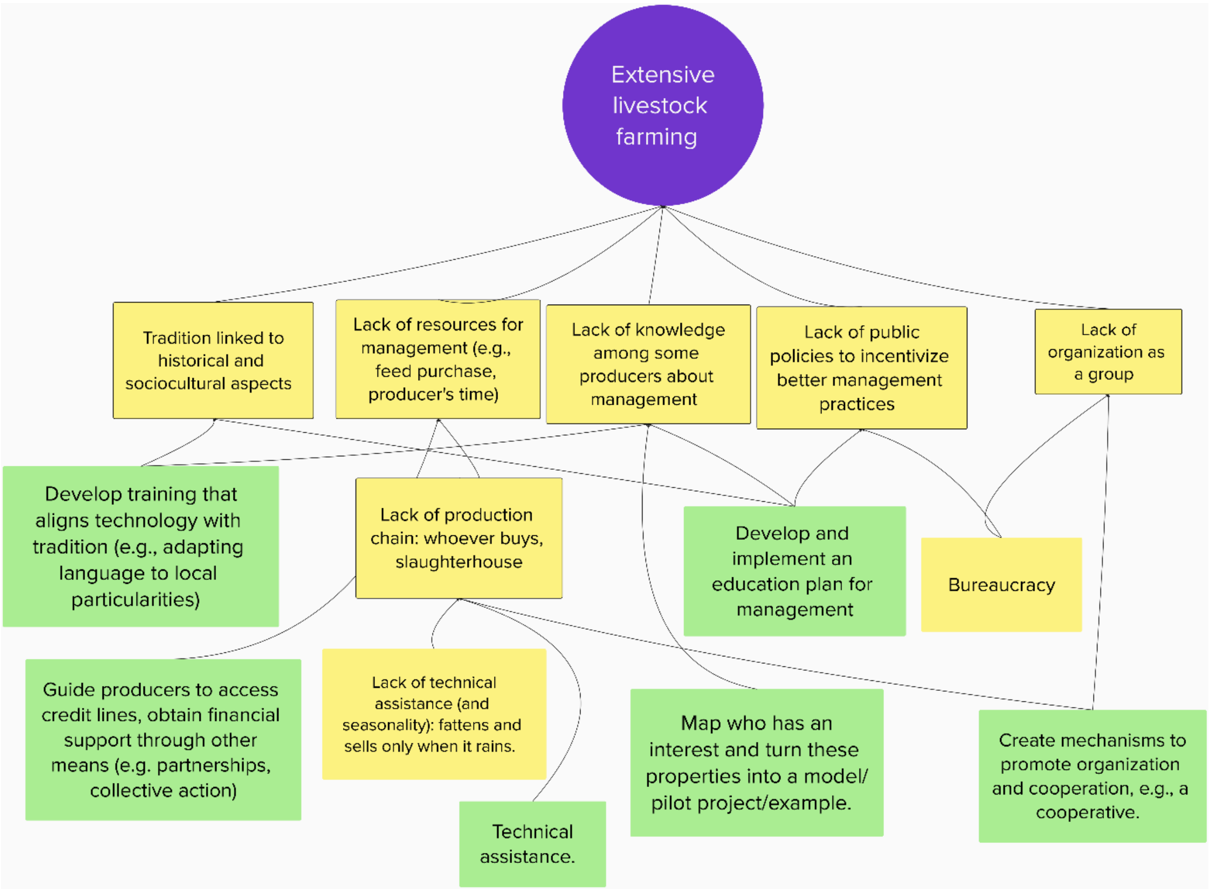

Extensive livestock farming
Tradition linked to historical and sociocultural aspects
Lack of resources for management (e.g., feed purchase, producer's time)
Lack of knowledge among some producers about management
Lack of public policies to incentivize better management practices
Lack of organization as a group
Develop training that aligns technology with tradition (e.g., adapting language to local particularities)
Lack of production chain: whoever buys, slaughterhouse
Develop and implement an education plan for management
Bureaucracy
Guide producers to access credit lines, obtain financial support through other means (e.g. partnerships, collective action)
Lack of technical assistance (and seasonality): fattens and sells only when it rains.
Map who has an interest and turn these properties into a model/ pilot project/example.
Create mechanisms to promote organization and cooperation, e.g., a cooperative.
Technical assistance.

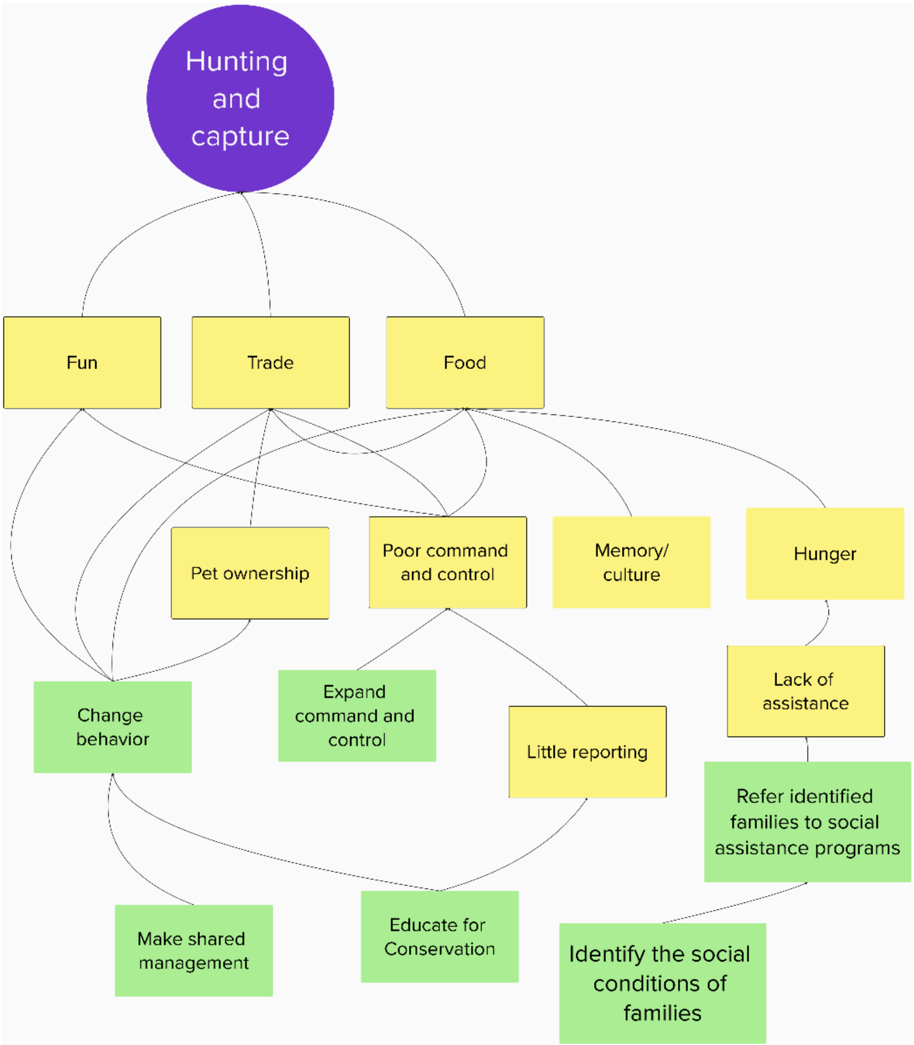

Hunting and capture
Fun
Trade
Food
Pet ownership
Poor command and control
Memory/ culture
Hunger
Change behavior
Expand command and control
Little reporting
Lack of assistance
Refer identified families to social assistance programs
Make shared management
Educate for Conservation
Identify the social conditions of families

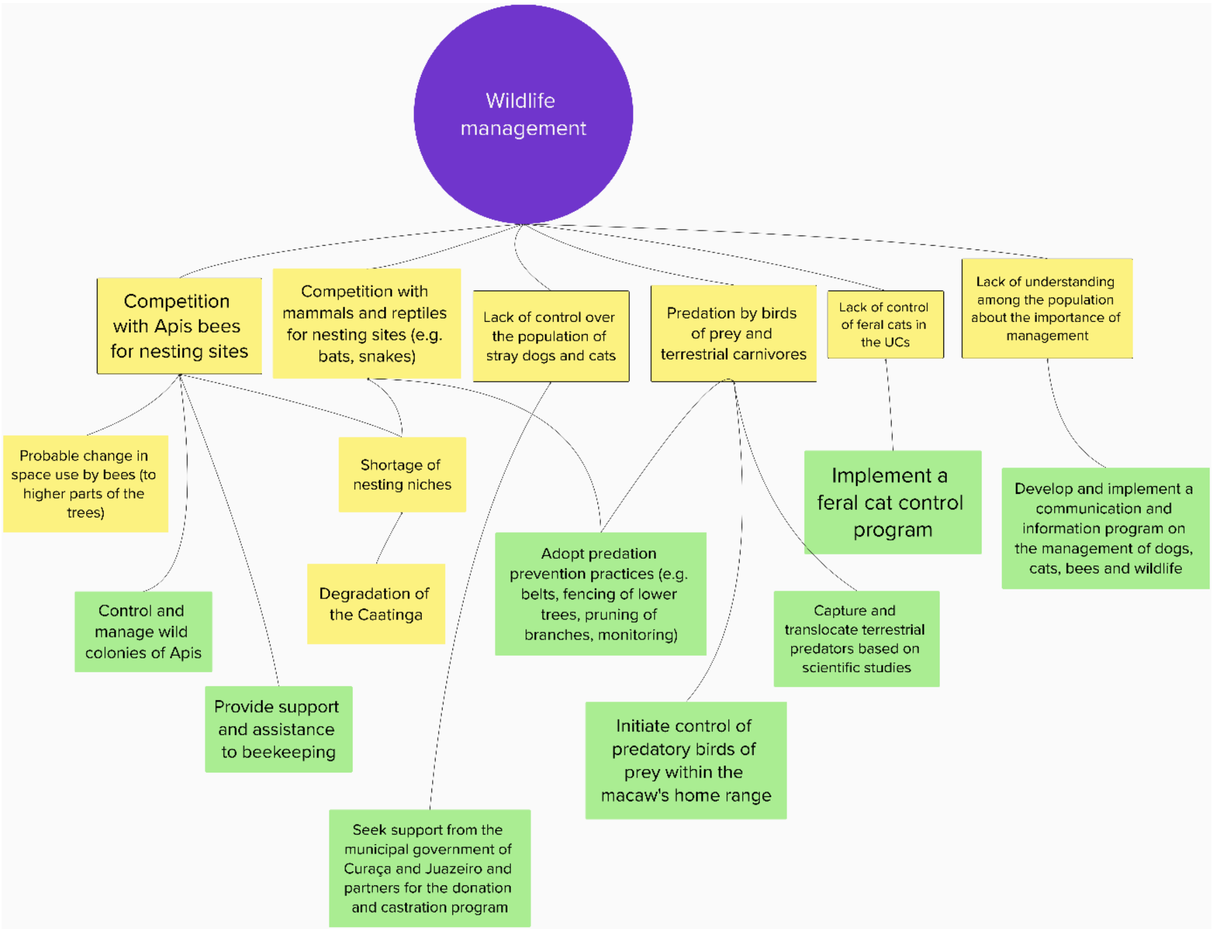

Wildlife management
Competition with Apis bees for nesting sites
Competition with mammals and reptiles for nesting sites (e.g. bats, snakes)
Lack of control over the population of stray dogs and cats
Predation by birds of prey and terrestrial carnivores
Lack of control of feral cats in the UCs
Lack of understanding among the population about the importance of management
Probable change in space use by bees (to higher parts of the trees)
Shortage of nesting niches
Implement a feral cat control program
Develop and implement a communication and information program on the management of dogs, cats, bees and wildlife
Degradation of the Caatinga
Adopt predation prevention practices (e.g. belts, fencing of lower trees, pruning of branches, monitoring)
Control and manage wild colonies of Apis
Capture and translocate terrestrial predators based on scientific studies
Provide support and assistance to beekeeping
Initiate control of predatory birds of prey within the macaw's home range
Seek support from the municipal government of Curaça and Juazeiro and partners for the donation and castration program

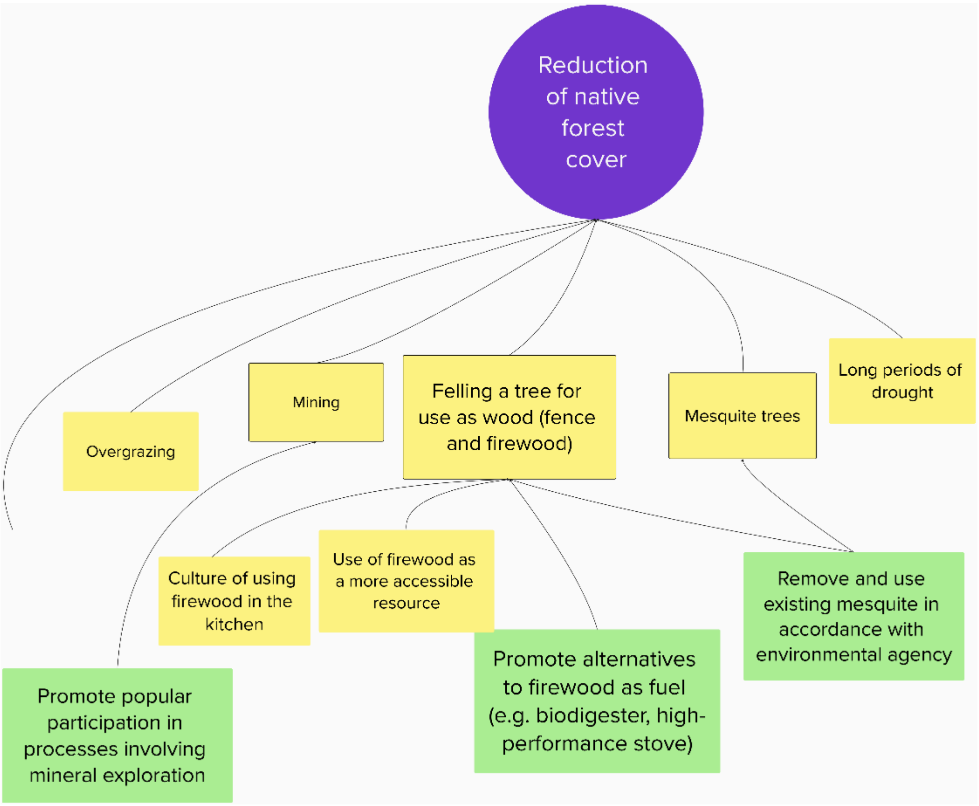

Reduction of native forest cover
Mining
Felling a tree for use as wood (fence and firewood)
Mesquite trees
Long periods of drought
Overgrazing
Culture of using firewood in the kitchen
Use of firewood as a more accessible resource
Remove and use existing mesquite in accordance with environmental agency
Promote alternatives to firewood as fuel (e.g. biodigester, high-performance stove)
Promote popular participation in processes involving mineral exploration

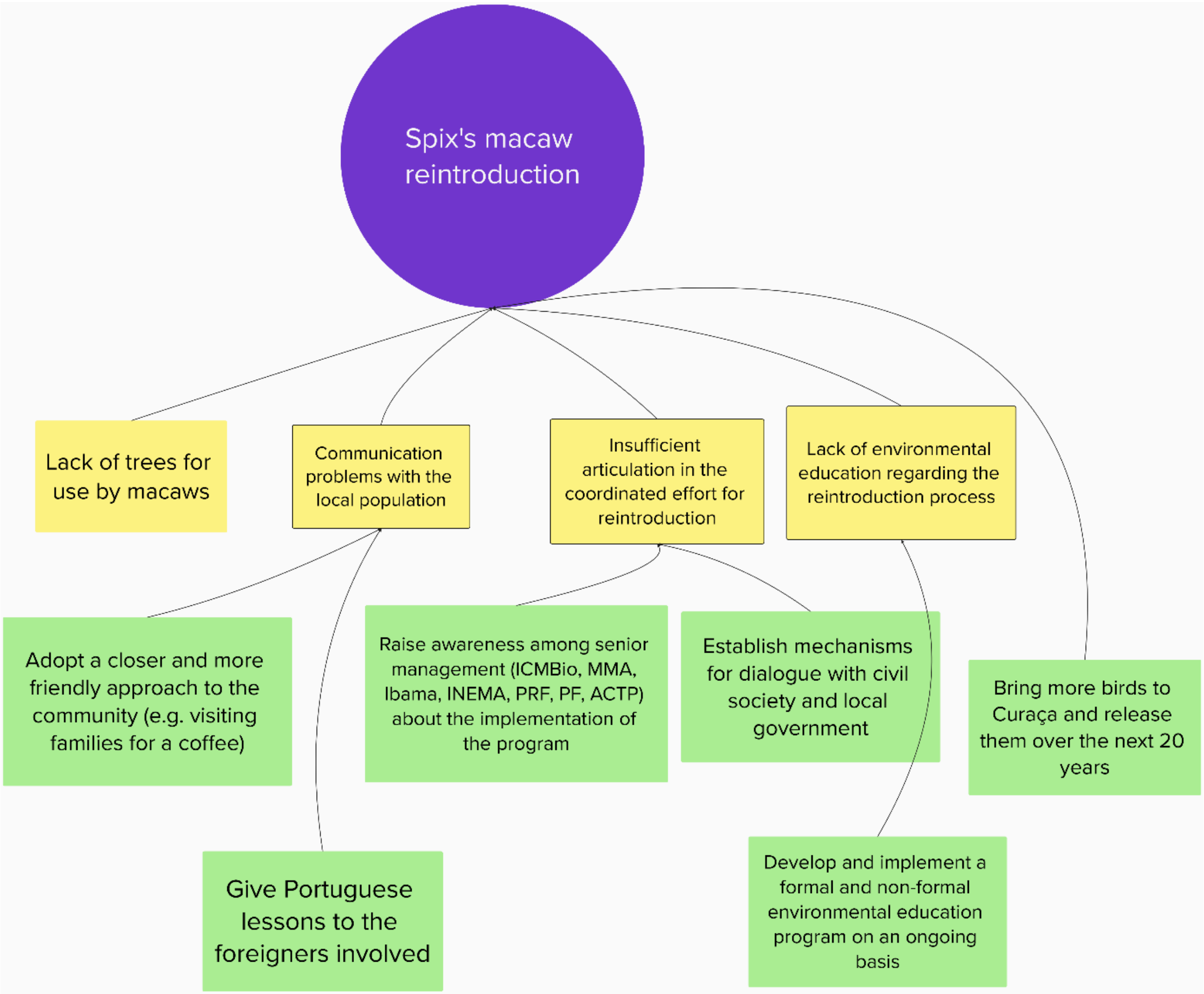
Spix's macaw reintroduction
Lack of trees for use by macaws
Communication problems with the local population
Insufficient articulation in the coordinated effort for reintroduction
Lack of environmental education regarding the reintroduction process
Adopt a closer and more friendly approach to the community (e.g. visiting families for a coffee)
Raise awareness among senior management (ICMBio, MMA, Ibama, INEMA, PRF, PF, ACTP) about the implementation of the program
Establish mechanisms for dialogue with civil society and local government
Bring more birds to Curaça and release them over the next 20 years
Give Portuguese lessons to the foreigners involved
Develop and implement a formal and non-formal environmental education program on an ongoing basis
