# Supplementary material for: Coexistence and habitat restoration planning for the reintroduction of Spix's macaw
Source: Conserv Biol. 2025 Jul 9;39(6):e70105. doi: 10.1111/cobi.70105 (PMC12658936; doi:10.1111/cobi.70105)
Supplement: Supplementary file 3 — Supporting Information [file COBI-39-e70105-s003.pdf]

**“COEXISTENCE AND HABITAT RESTORATION PLANNING FOR THE REINTRODUCTION OF THE SPIX’S MACAW" – Appendix S3**

**Action Plan for Coexistence Matrix**

| | Action | Output | First step |
|---|---|---|---|
| 1 | Provide legal and technical support for regularization | Regularized documentation | Completing the checklist, legal analysis |
| 2 | Disseminate experiences in all sectors (communities, academia, companies) | Photos, videos and materials | Scheduling visits |
| 3 | Conduct medium/long-term research and establish protocols for reforestation | Planting protocol | Planning meeting with the main questions to be answered |
| 4 | Implement mechanisms for local community participation in defining techniques | Ensure community participation | Community meetings |
| 5 | Develop social technologies applied to reforestation (e.g. successive dams, contour lines) | Dams, contour lines | Identification of areas |
| 6 | Support land uses that are compatible with reforestation (e.g. use of pigpens, fencing) | List of management practices | Identifying land use practices compatible with ‘recaatingamento’, |
| 7 | Discuss and negotiate the contract model with interested parties | Contract model | Visits and meetings |
| 8 | Hold workshops/seminars on carbon credits | Workshops/Seminars | Planning, organization and mobilization |
| 9 | Create and implement municipal PES | Law | Gather stakeholders |
| 10 | Map and support ongoing ecosystem projects/practices | Ecosystemic projects | Identify ongoing projects |
| 11 | Develop and implement certification | Certificate/ Quality Seal | Identify/organize producers, community adaptation |
| 12 | Enable farmers to participate in food acquisition | Farmer Qualification/ Declaration | Identify/organize producers |
| 13 | Connect producers with CooperCUC | Marketing | Identify/organize producers |
| 14 | Provide ongoing training | EC Program/ Trained Producers and Producer Certification | Mobilize collaborators |
| 15 | Create a network for manure supply | Manure/ Supply Network, Producer Certification | Identify interested producers |
| 16 | Create a seed network | Seed Network, Producer Certification | Identify those interested in collecting native seeds |
| 17 | Promote nurseries | Native seedling nurseries | Identify locations with infrastructure and water; Acquire inputs; Make contacts to build partnerships. |
| 18 | Open public notice for ATER in agroecology | Call for proposals opened by the government | Promote the opening of the call for proposals |
| 19 | Map, recover, build structures for water storage (e.g. dams, cisterns) | Map of areas for recovery of water storage structures; | Visit and recognition of the area for diagnosis. |
| 20 | Develop training that aligns technology with tradition (e.g. adapting language to local particularities) | Training Workshops | Identify interested people, availability of people. |

| | | | |
|---|---|---|---|
| 21 | Guide producers to access credit lines, obtain financial support through other means (e.g. partnerships, collective efforts) | Consulting group | Invite associations (Launch proposals for associations) |
| 22 | Technical assistance | Implementation of more efficient semi-intensive/intensive goat and sheep farming. | Identify and listen to interested parties. |
| 23 | Map who is interested and turn these properties into a model/pilot project/example | Identification of interested parties | Identify and listen to interested parties. |
| 24 | Develop and implement an education plan for management | Education plan | Planning meeting with partner institutions |
| 25 | Create mechanisms to foster organization, cooperation. E.g. cooperative | Cooperative | Identification of the production chain |
| 26 | Create/support community-based ecological tourism | Tourism Plan | Mapping of tourist attractions. |
| 27 | Define and publicize the municipality's tourist route | Tourism Route | Mapping and promotion of tourist attractions. |
| 28 | Develop and implement a multi-year municipal policy for community-based tourism | Multi-Year Municipal Policy | Coordination between public authorities and communities |
| 29 | Education for Conservation | Environmental Education | Partners meeting |
| 30 | Shared management | Management Unit | Partners meeting |
| 31 | Expand inspection actions | Intensification of monitoring | More frequent presence of inspection bodies in communities, regardless of complaints. |
| 32 | Refer identified families to social assistance programs | Referral of Families | Identify families |
| 33 | Control and manage wild Apis colonies | Control and Management | Preparation of management plan. |
| 34 | Provide support and assistance to beekeeping | List of interested parties in receiving support for beekeeping | Map stakeholders; mobilize collaborators |
| | | Boxes and bees provided | Search for resources and suppliers |
| | | Trained producers | Develop course and identify partners |
| 35 | Seek support from the municipal government of Curaça and Juazeiro and partners for a donation and neutering program | Castration and donation project | Meeting with municipal departments and collaborators and write the project |
| | | Bill created | Write the project and contact the government |
| 36 | Initiate control of predatory birds of prey within the macaw's home range | Control of the population of raptors and monitoring of control | Gather collaborators to begin control and monitoring |
| 37 | Adopt predation prevention practices (e.g., belts, fencing of lower trees, pruning of branches, monitoring) | Prevention practices adopted | Ariane contact the person responsible for CENAP to list prevention measures and adopt them |
| 38 | Capture and translocate terrestrial predators based on scientific studies | Survey of terrestrial predators to be translocated | Contact Claudia and Camile; identify the person responsible for the survey |
| 39 | Implement a feral cat control program | Survey of the population of feral cats | Contact UNIBRAS and plan a survey and control |

| | | | |
|---|---|---|---|
| | | within the UCs carried out | together with ICMBio and Bluesky |
| | | Feral cats controlled within the UCs | Survey feral cat populations in the UCs, identify who will control them and how, and the destination |
| | | Possible new populations of feral cats within the monitored UCs | Identify the person responsible for monitoring |
| 40 | Develop and implement a communication and information program on the management of dogs, cats, bees and wildlife | Teams of information multipliers formed | Identify multipliers, organize the information, multiply |
| 41 | Encourage public participation in processes involving mineral exploration | Popular participation | Contact representatives of collaborating agencies and request community participation in decisive meetings |
| 42 | Promote alternatives to firewood as fuel (e.g. biodigester, high-performance stove) | Community members trained<br><br>Guide for the production of alternative fuels | Bluesky should contact the Perene Institute (high-performance stove), and seek collaborators to develop the biomanager identify partners to produce the guide |
| 43 | Remove and use existing mesquite trees in accordance with environmental agency | Removal of mesquite from planting areas | Define the areas |
| 44 | Adopt a closer and more friendly approach with the community (e.g. visiting families for a coffee) | Community outreach | Visits |
| 45 | Give Portuguese lessons to foreigners | Classes held | Hire a teacher |
| 46 | Raise awareness among senior officials (ICMBio, MMA, Ibama, INEMA, PRF, PF, ACTP) to implement the program | Letter of appeal from society about the importance of continuing the project<br>Technical cooperation agreement with ACTP and ICMBio renewed | Prepare manifesto for signature on March 3rd<br><br>Send correspondence to ICMBio |
| 47 | Establish mechanisms for dialogue with civil society and local government | Periodic meetings (council of the units) | Bluesky to be present at the March 3rd meeting |
| 48 | Develop and implement a formal and informal environmental education program on an ongoing basis | Environmental education program implemented | Flávia to contact collaborators to initiate proposal |
| 49 | Bring more birds to Curaçao and release new ones over the next 20 years | 20 birds released per year | Meeting with ICMBio |
